# Thoracoscopic thymectomy for juvenile myasthenia gravis

**DOI:** 10.1007/s00383-019-04441-0

**Published:** 2019-02-07

**Authors:** Aimee G. Kim, Sydney A. Upah, John F. Brandsema, Sabrina W. Yum, Thane A. Blinman

**Affiliations:** 1 Division of Pediatric General, Thoracic and Fetal Surgery, Children’s Hospital of Philadelphia, 34th Street and Civic Center Boulevard, Philadelphia, PA 19104 USA; 2 Division of Neurology, Children’s Hospital of Philadelphia, 34th Street and Civic Center Boulevard, Philadelphia, PA 19104 USA

**Keywords:** Minimally invasive surgery, Thoracoscopy, Thymectomy, Juvenile myasthenia gravis

## Abstract

**Purpose:**

A randomized controlled trial of thymectomy in myasthenia gravis demonstrated improved clinical outcomes in adults, but data surrounding juvenile cases, especially those treated with minimally invasive approaches, are limited. Here, we review our experience with thoracoscopic thymectomy for juvenile myasthenia gravis (JMG) in the largest cohort to date.

**Methods:**

All cases of thymectomy for JMG in a single tertiary referral center between 2007 and 2018 were reviewed (*N* = 50). Patients underwent left thoracoscopic approach with extended dissection and without use of monopolar energy. Demographics, diagnostic criteria, and clinical classification, as well as surgical data were collected. Clinical status and medications were reviewed in follow-up.

**Results:**

The mean age at surgery was 10.5 ± 0.8 years. Ocular disease and generalized disease each comprised half of the cohort. No patients suffered complications or increased risk of morbidity or mortality with thymectomy. At any interval of follow-up through 3.5 years, 49.8% of patients were improved compared to their pre-operative presentation, and there was a significant trend towards decreased steroid use.

**Conclusion:**

Thoracoscopic thymectomy is a safe treatment for juvenile myasthenia gravis in pediatric patients over a wide range of ages, body masses, and symptoms. Our experience adds evidence that pediatric patients likely benefit from thymectomy with improved clinical status and reduced medications.

## Background

Myasthenia gravis (MG) is an autoimmune disease characterized by the production of antibodies against acetylcholine receptors (AChR) in the neuromuscular junction. Patients experience debilitating fluctuating weakness of various muscle groups, e.g. ocular, facial, limb, swallowing, or respiratory, and symptoms are exacerbated by activity and stress. In childhood, the disease presents in two forms, transient neonatal MG and juvenile MG (JMG). Twenty percent of babies born to women with MG exhibit transient neonatal MG with hypotonia and feeding difficulties that usually resolve within 2 months with supportive therapy [1]. JMG comprises a subset of patients exclusive of the transient form in whom symptom onset occurs before 18 years of age.

JMG is diagnosed by clinical presentation supported by laboratory assessment of antibodies (predominantly AChR, but also muscle-specific kinase (MuSK)) and neuromuscular testing to evaluate for electrodecrement on repetitive nerve stimulation. There is no evidence to indicate that pathophysiology differs between JMG and adult onset MG. Retrospective and longitudinal studies in the pediatric population have shown children with MG are more likely to present with ocular symptoms, and the estimated frequency of generalized disease ranges from 29 to 75%, which is less than is reported in the adult population [2]. However, ocular disease may progress to generalized symptoms in 30–50% of pediatric/adolescent patients [3]. Spontaneous remission may be more likely in JMG at 14.3–45% compared to adult-onset disease [4, 5]. Consensus on management of JMG is further complicated by differences in presentation, disease course, and response to treatment as related to gender, race, age of onset, and antibody status [6–8].

This variability compounded with the low incidence of JMG and the ethical challenges of pediatric trials have thus far prohibited large-scale prospective studies and development of strong evidence-based practice guidelines.

Treatment for JMG broadly follows adult MG guidelines. First-line therapy is with acetylcholine esterase inhibitors (AChEI), which may suffice for ocular-only disease. Immunosuppressive agents and immunomodulating therapy are added for refractory or progressing disease. With development of generalized muscle weakness, a multimodal approach is indicated including AChEI, immunosuppressive agents, immunomodulating therapy, and surgery [9].

Thymectomy offers improved chance of remission, reduction in medical therapy and its complications (e.g. from long-term steroid use), and reduced risk of progression of disease. These effects are well described in the adult literature with evidence suggesting that MG patients after thymectomy are more likely to achieve symptom-free, medication-free remission. Those with severe and generalized symptoms and those undergoing “early” thymectomy may have a better chance of remission [10]. A recent multicenter randomized trial in adults comparing thymectomy with steroid therapy versus steroid therapy alone demonstrated superior clinical outcomes (lower Quantitative Myasthenia Gravis score, decreased medication usage, and fewer hospitalizations) in the thymectomy cohort over a 3-year period [11]. Different approaches (transsternal, transcervical, thoracoscopic) have been investigated, and generally show similar efficacy [12, 13].

In the pediatric literature, multiple retrospective studies have supported thymectomy for peri-pubertal and post-pubertal AChR-positive JMG with moderate to severe symptoms. A recent systematic review of 16 studies recorded post-operative improvement in JMG severity in 77% of patients, including 29% with complete sustained remission [14]. Similar to adults, “early thymectomy” (performed within a year of diagnosis) resulted in higher remission rates compared to “late thymectomy” [15, 16]. Support for thymectomy in JMG includes minimizing the adverse effects of immunosuppression such as growth retardation and returning patients to baseline neurological function as soon as possible to prevent potential developmental delays. Thoracoscopic thymectomy offers significant benefits with reports of few complications, shorter hospital stays, and better aesthetic outcomes, but raises the risk of a potentially incomplete resection [17–19].

Here we present our experience with a minimally invasive surgical approach for thymectomy in the pediatric population.

## Methods

Institutional Review Board approval (#14-011298) was obtained for the study. A retrospective review of all patients undergoing thymectomy for the treatment of myasthenia gravis was performed at a single tertiary referral center between 2007 and 2018 (*N* = 50). Medical records were examined for patient demographics, clinical presentation, medical management, time to surgical intervention, and indication for surgery, as well as operative and post-operative data. Patients for whom pre-operative and follow-up visits were available had their charts reviewed and abstracted for inpatient or outpatient visits at half-year intervals. Data were not uniformly available for all post-operative intervals in this retrospective review; visits were assigned to the half-year time-point to which it was closest (within 3 months). Patients’ clinical state was compared to their pre-operative condition. Disease severity was assessed as documented in the physical exam performed by Neurologists or Ophthalmologists and graded per criteria from the Myasthenia Gravis Foundation of America Postintervention Status (MGFA-PIS) and the DeFilippi classification. The MGFA-PIS defines an “improved (I)” change in status compared to pre-intervention as “A substantial decrease in pretreatment clinical manifestations or a sustained substantial reduction in MG medications as defined in the protocol” [20]. We defined a “substantial reduction in MG medications” as a 50% decrease in medication dose. Under the DeFilippi designations, Classes 1–3 all describe a favorable clinical state, with 1 being “Complete remission off all medications”, 2 for “Asymptomatic on decreased medications”, and 3, “Improved, with decreased symptoms or decreased medications” [21]. Weight-adjusted daily medication doses were calculated for all patients on steroids (prednisone or prednisolone) and for whom weights were documented at the appropriate follow-up visit.

Descriptive statistics were utilized throughout to summarize demographic, perioperative, and outcome data, presenting means ± standard error of the mean (SEM) unless otherwise indicated. A Kaplan–Meier event curve was generated using MGFA-PIS “Improved” status as the “event” captured at the time of final follow-up and calculating cumulative probabilities. One-way ANOVA and linear regression analyses were applied to evaluate trends in medication dosing throughout follow-up. All statistical analyses were performed using Microsoft Excel and GraphPad Prism version 7.0 for Mac. Statistical significance was interpreted at *p* < 0.05.

### Surgical technique

**Fig. 1:**
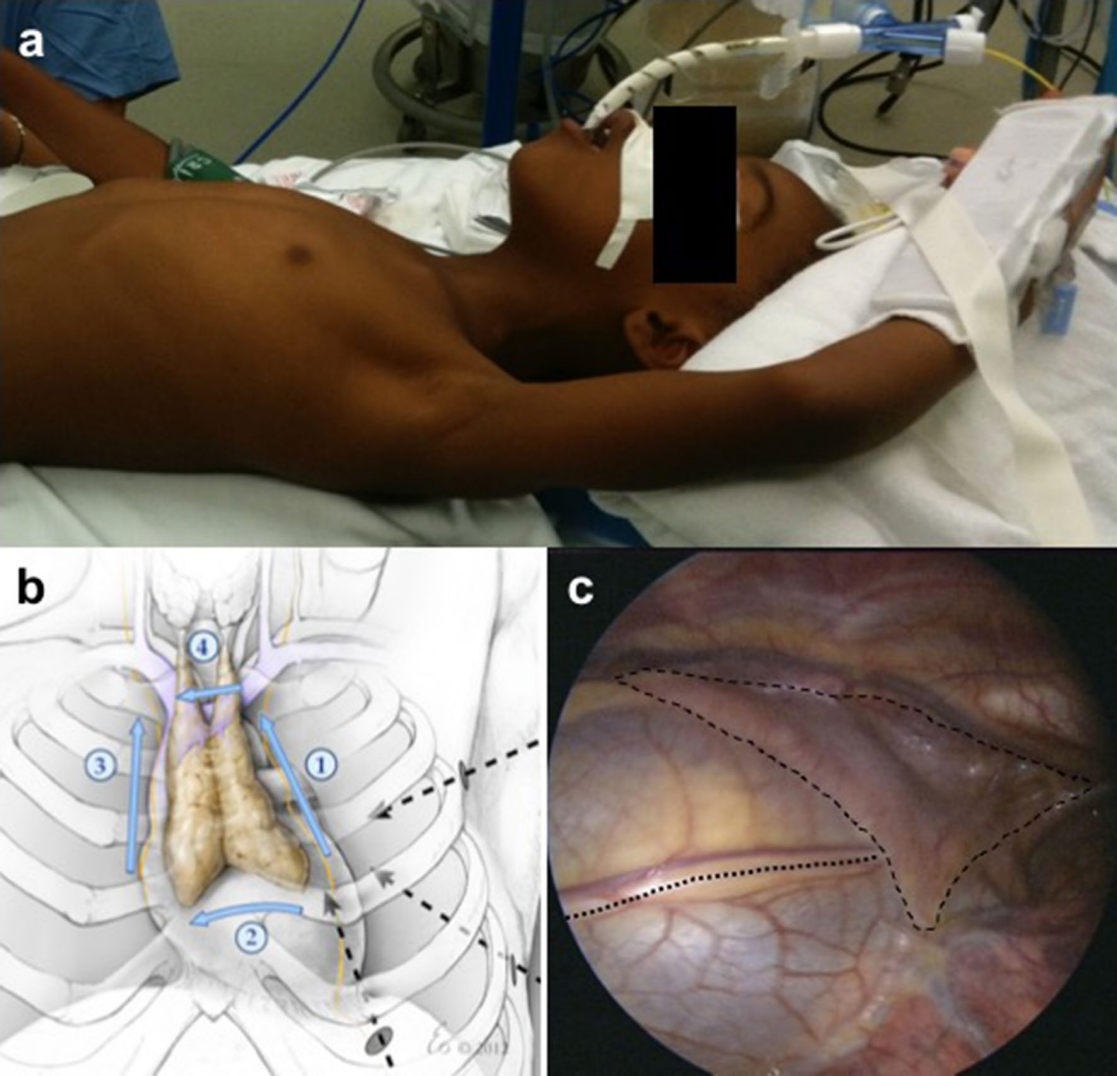
**a** Patient positioned with bump under the left chest and left arm gently abducted. **b** Diagram depicting port placement and operative approach. **c** Laparoscopic view of the thymus (dashed line) and the course of the phrenic nerve (dotted line)

Patients were positioned supine with a small bump under the left chest, and the left arm gently abducted and padded (Fig. 1a). Single lung ventilation was not used; instead, low pressure CO_2_ insufflation (< 6 mmHg) and PEEP < 2 allowed functional isolation while gravity effectively retracted the lung. Three 5-mm trocars were placed using a blunt or optical technique (Fig. 1b). The left phrenic nerve was identified and preserved. All dissections were blunt, cold, sharp, or with the Ligasure™ (Covidien, Dublin, Republic of Ireland) bipolar device. After opening the pleura over the thymus and allowing the phrenic nerve to fall posteriorly with the associated pleura, the inferior poles were freed, taking care to include associated pericardial fat. The largely avascular planes above and below the thymus were also developed mostly with blunt dissection, working across the mediastinum. The contralateral pleura can be seen as a white line when the thymus is retracted toward the left hemi-thorax, and the plane between thymus and right pleura was gently developed bluntly. Then when the inferior poles were largely free, the innominate vein was identified and skeletonized, dividing the one or two draining veins from the thymus using the Ligasure. Occasionally, small feeding arteries can be identified in the loose tissue anteriorly, and these were also divided with the Ligasure. The superior horns were then traced well up into the neck until they tapered down to thin connective tissue often with lymphatic tissue associated. These poles were also divided with the Ligasure. The thymus was retrieved using an endoscopic specimen bag. The anterior mediastinum was inspected to ensure that it had been skeletonized and no residual thymus remained. Residual CO2 was evacuated with a chest tube pulled before closing the last trocar incision with a static positive pressure breath administered by the anesthesiologist.

## Results

### Demographics

The mean age at diagnosis was 8.8 years ± 0.8 (range 1–17 years) (Table 1). The majority of patients were female (72.0%). Just over half of patients (54.5%) exhibited generalized symptoms. The majority were on pyridostigmine and steroids (prednisone or prednisolone), and half of patients were on both. Approximately, a third of patients (34.1%) were on chronic intravenous immunoglobulin (IVIG) therapy.

**Table 1:** Demographics

Age at diagnosis (*n* = 49)	8.8 ± 0.8
≤ 10 years old (%)	55.1
11–18 years old (%)	44.9
Gender	
Female (%)	72.0
Male (%)	28.0
Seropositivity	
AchR (%) (*n* = 48)	62.5
Classification at pre-op (*n* = 44)	
Ocular (%)	45.5
Generalized (%)	54.5
Management at pre-op (*n* = 44)	
Pyridostigmine (%)	77.3
Steroids (%)	65.9
Chronic IVIG (%)	34.1
Plasmapheresis (%)	9.1

The two most common indications for thymectomy were failure of medical therapy and generalized/bulbar symptoms (Table 2). The former indication included patients in whom symptoms were not controlled on escalating medical therapy and those in whom steroids could not be weaned.

**Table 2:** Indications for thymectomy

Failure of medical therapy (%)	27.3
Systemic/bulbar symptoms (%)	27.3
Increase chance of remission/reduce meds (%)	22.7
Suspected thymic abnormality (%)	11.4
Reason not specified (%)	11.4

### Perioperative data

The mean age at surgery was 10.5 years ± 0.8 (range of 3–17 years) (Table 3). Consistent with the wide age range, patients exhibited a range of weights and a mean body mass index (BMI) of 22.3 ± 1.0 (range 13.0–49.6). On average, patients were on medical therapy prior to surgery for 19.6 months ± 4.2 (*n* = 49, range of 0–168 months), and 51% of patients had an “early thymectomy,” defined as occurring within 1 year of diagnosis.

**Table 3:** Perioperative and surgical data

Age at surgery (*n* = 50)	10.5 ± 0.8
BMI at surgery (*n* = 49)	22.3 ± 1.0
Time to thymectomy (months) (*n* = 49)	19.6 ± 4.2
Early thymectomy (%)	51.0
Pre-op conditioning with IVIG (%)	42.0
Operative time (min)	104 ± 3.5
Estimated blood loss (mL)	2.9 ± 0.3
Post-operative ICU admission (%)	18.0
Complications (%)	0
Post-operative stay (days)	1.2 ± 0.1
Histopathology	
Normal (%)	52.0
Thymic hyperplasia (%)	30.0
Thymoma (%)	0
Other (%)	18.0

### Surgical outcomes

The mean operative time was 104 min ± 3.5 (range of 62–163 min). Patients stayed in the hospital a mean of 1.2 days ± 0.1 after surgery with 86% of patients being discharged on post-operative day 1. Nine patients (18%) were admitted to the intensive care unit (ICU) post-operatively. There were no complications: no conversions to thoracotomy, no median sternotomy, no significant blood loss, no transfusions, no prolonged intubations, and no re-intubations. No thymomas were identified in any pathological specimen. Approximately half of specimens were normal on histopathology (52%) and less than one-third (30.0%) displayed thymic hyperplasia.

### Clinical outcomes

**Fig. 2:**
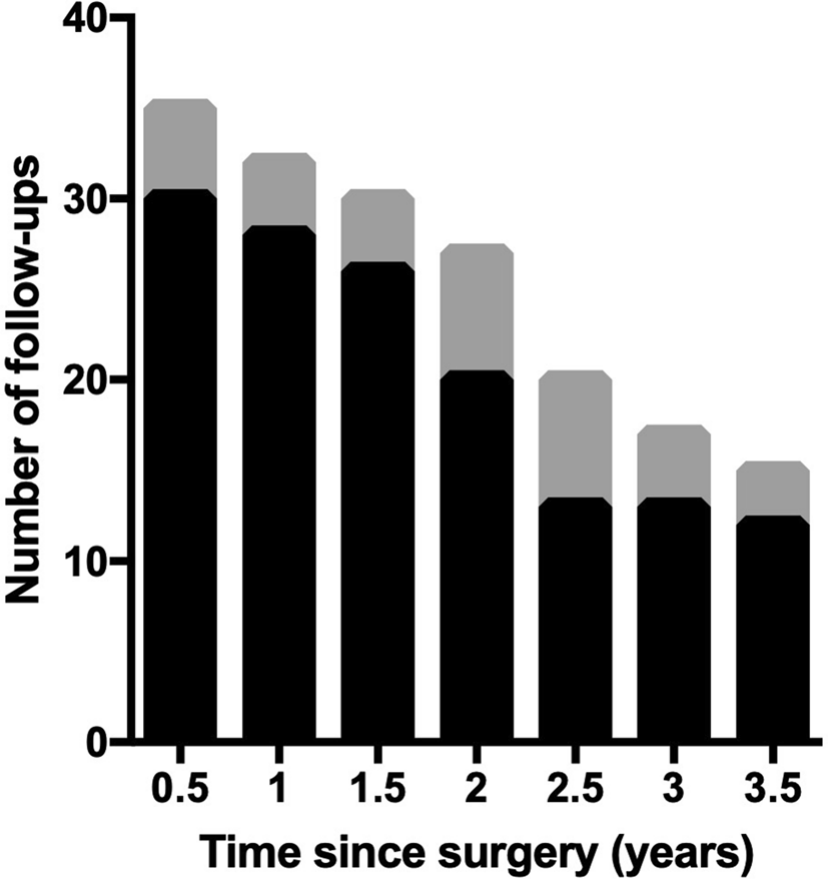
An average of 79.3% of patients in whom pre- and post-operative data were available presented for follow-up at any given 6-month interval between 0.5 and 3.5 years after thymectomy; the gray bars depict the number of patients due for follow-up (excluding those who relocated or transitioned to adult care at ≥ 18 years of age), the black bars depict the number of those that presented for follow-up

Follow-up data were available for 41 patients and ranged from 1 to 11 years post-operatively. Thirty-five patients had pre-operative and follow-up data, with a mean of 79.3% of patients returning for follow-up through 3.5 years, excluding those who re-located or transitioned to adult care at ≥ 18 years of age (mean follow-up duration 37.9 ± 4.2 months) (Fig. 2). After 3.5 years, the percentage of patients with follow-up data acutely declined to less than 50%, resulting in a sample size too small for reliable statistical assessment of outcomes.

**Fig. 3:**
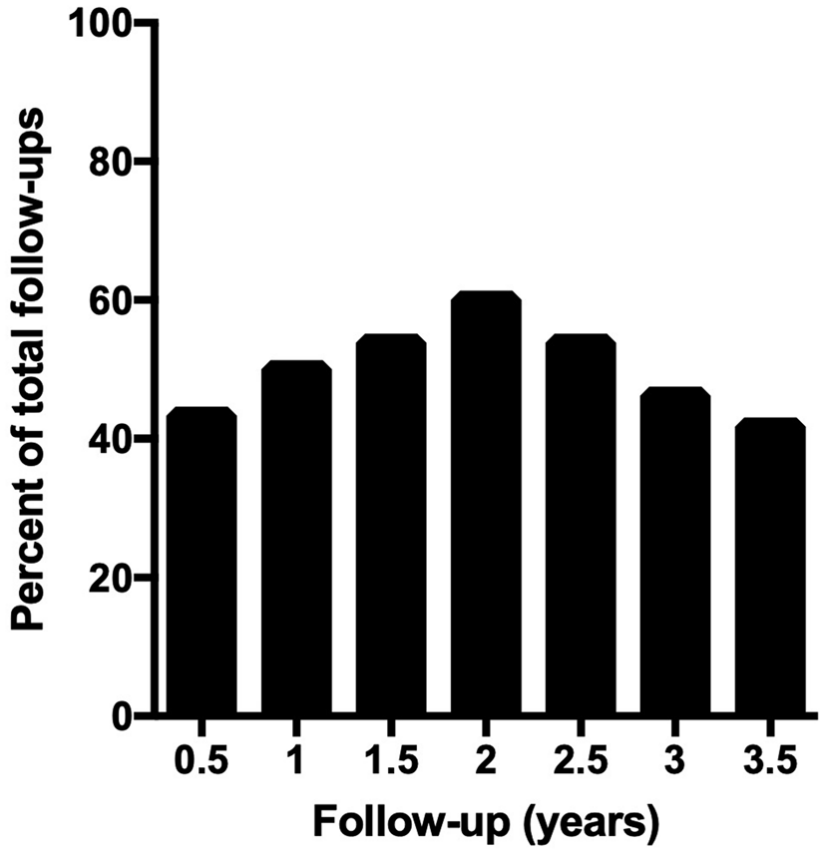
At any given half-year follow-up between 6 and 42 months after surgery, a mean of 49.8% of patients were “improved” compared to their pre-operative status, as defined by the MGFA-PIS classification with substantial decrease in clinical symptoms or 50% reduction in medications

**Fig. 4:**
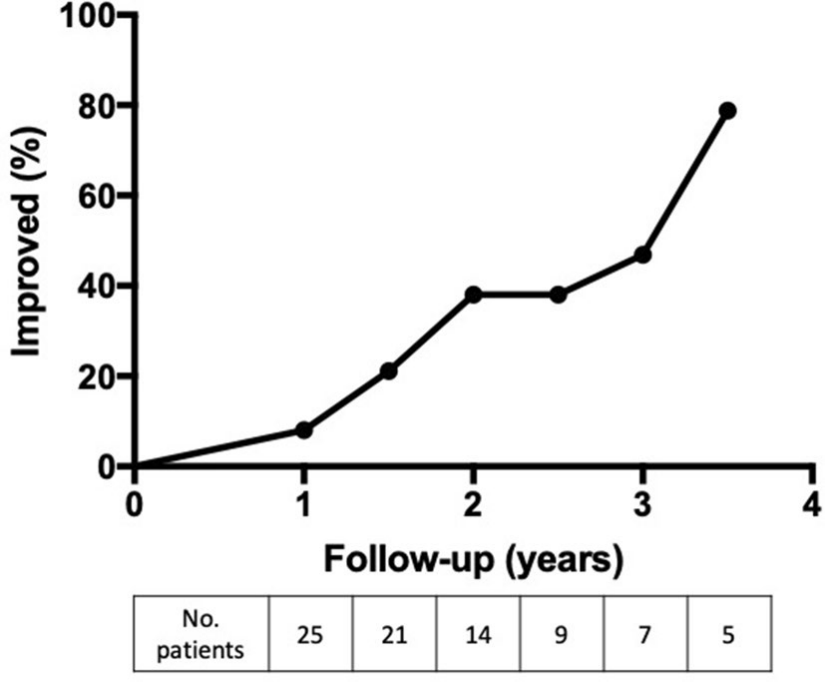
Kaplan–Meier event curve for “Improved” clinical status by MGFA-PIS classification at last follow-up visit with the number of patients “at risk” at each time-point depicts increasing cumulative probability of “improved” clinical status after thymectomy

A mean of 49.8% of patients showed an “Improved” MGFA-PIS change in status compared to prior to surgery through 3.5 years of post-operative follow-up (Fig. 3). Using the DeFilippi classification over the same period, a mean of 61.9% of patients scored within classes 1 to 3, demonstrating improvement either by clinical symptomatology or decreased medication requirements (data not shown). There was no statistically significant difference in the percentage of “Improved” patients when the cohort was stratified for ocular versus generalized type, age at thymectomy, or timing of thymectomy. A Kaplan–Meier event curve looking specifically at those with follow-up through 3.5 years after thymectomy at their last recorded visit shows an increasing cumulative probability of “Improved” status (Fig. 4).

**Fig. 5:**
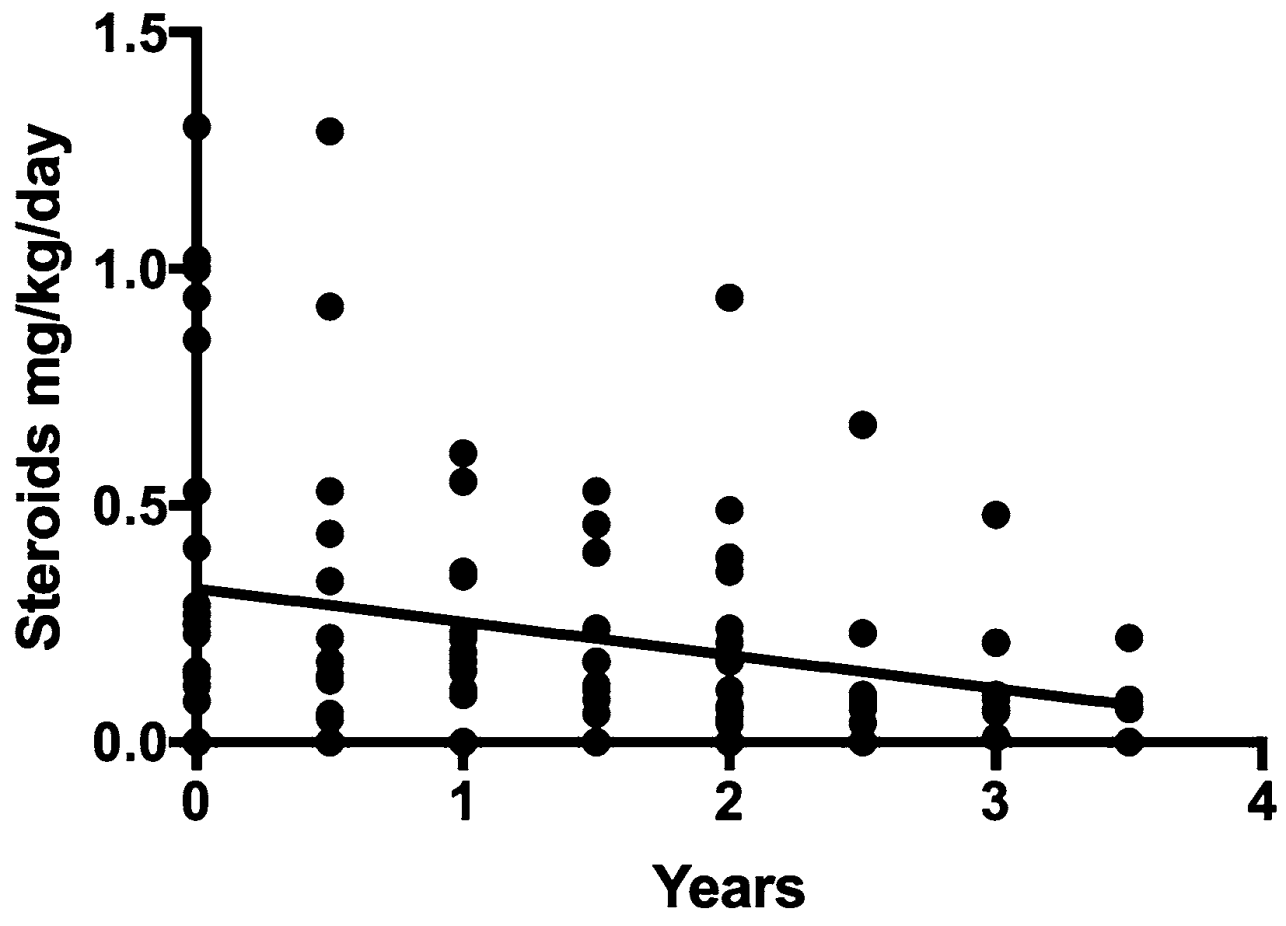
Weight-adjusted total daily steroid intake (mg/kg/day) decreased significantly over 3.5 years of follow-up on linear regression analysis

Of the 35 patients in whom pre- and post-operative data are available, none were on any non-steroid oral immunosuppressive agent at the time of surgery. Eight patients were initiated on an agent post-operatively (seven on azathioprine and one on mycophenolic acid). All but one patient were on an agent at the last available follow-up visit. Looking specifically at weight-adjusted daily total steroid dose, there was no statistically significant difference by one-way ANOVA over 3.5 years of follow-up. On linear regression analysis, however, total daily steroid intake (*p* = 0.0027) decreased significantly over the same follow-up period (Fig. 5). There was no significant difference in the proportion of patients on pyridostigmine therapy at the time of surgery compared to that in follow-up.

## Discussion

This series reports on the largest cohort of thoracoscopic pediatric thymectomy cases with 79% adherence to long-term post-operative follow-up, providing more robust evidence than has been published to date. In a cohort of variable ages, disease severities, and timing to operative intervention, there were no adverse events and the majority of patients were discharged 1 day after surgery. At any given half-year follow-up interval between 6 months and 3.5 years after surgery, half of patients demonstrated clinical improvement compared to their pre-operative state, either with decreased symptoms and/or medications. Finally, we report a significant trend towards decreased weight-based steroid dosing after thymectomy. This study adds substantial weight of evidence for the safety of thoracoscopic thymectomy and its place in the clinical management of JMG.

The role of thymectomy for adult MG, already a well-established treatment modality, was recently validated in a randomized controlled trial (RCT) as superior treatment compared to steroid therapy alone in achieving symptom resolution, decreasing total steroid dose, and enhancing quality of life [11]. Studies in the JMG population are far less robust, though multiple retrospective analyses have shown both open and minimally invasive approaches to be safe and effective in children [17, 18, 22]. Thoracoscopic series are fewer in number, but suggest benefits of decreased estimated blood loss, shorter hospital stays, and improved cosmesis [23, 24]. Madenci et al. in 2017 published a systematic review of the literature from 2000 to 2016 regarding thymectomy for the treatment of JMG [14]. In 16 retrospective studies including 1131 pediatric patients of which 43% underwent thymectomy, 77% exhibited improvement in JMG severity after thymectomy with 2–5 years of follow-up. The majority of studies reported on the open transsternal approach (82% of patients) and four studies compared surgical approaches, with insufficient power for statistical comparison. Few studies compared outcomes of patients who underwent thymectomy against those that did not—and their findings were mixed.

In our cohort, a smaller percentage of patients exhibited “improved” clinical status compared to what is reported by Madenci et al. (50% versus 77%). We attribute this difference to the application of a more stringent definition of “clinical improvement.” Change in clinical status was evaluated according to the MGFA-PIS classification system and relied on the documented physical exam of the treating neurologist or ophthalmologist and/or a 50% decrease in medication dosage documented at the follow-up visit. When evaluating the same cohort by the DeFilippi system, which does not use objective measures for grading, we also observed a higher mean percentage of patients designated as “improved” compared to that determined by MGFA-PIS. Despite the increased sample size, our study is insufficiently powered for statistical demonstration of remission, defined by the MGFA as “no symptoms or signs of MG for at least 1 year and… no therapy for MG during that time” [20]. Remission patterns in JMG have been shown to wax and wane, significantly affected by external stressors and illness, but with an overall trend towards greater improvement further out from surgery [23, 25]. Longitudinal follow-up of this population will be required to determine true effectiveness of operative intervention here.

Another significant finding in the data involved decreasing medications in patients after thymectomy. Medical management of JMG long term is not benign, especially in the pediatric patient, as therapy can result in side effects both inconvenient and detrimental to growth and development during a critical period. Over a fifth of patients underwent surgery to reduce medications; though this result has been observed in the pediatric literature, no statistically significant difference had yet been demonstrated in JMG. We found weight-adjusted total daily steroid intake declined significantly over the course of follow-up on linear regression analysis. This result is notable in light of the significant adverse effects of steroids on development and growth. As well, our data suggest a larger sample size and longer follow-up in a pediatric RCT may recapitulate the findings in the adult RCT in which the time-weighted average prednisone dose was significantly lower in patients after thymectomy compared to those on medical management alone.

In the adult literature early thymectomy for ocular MG has been promoted to prevent onset of generalized symptoms [26]. Thymectomy for ocular MG in children is controversial. About 1/3 of ocular MG cases develop systemic symptoms but most progress through a relatively benign course with medical management. Especially in the pre-pubescent group, generalization seems to occur at a much lower rate and spontaneous remission appears more common [27]. In our series, 45.5% exhibited only ocular symptoms at the time of surgery and none of these patients converted to generalized disease in follow-up. Further, in subgroup analysis of ocular versus generalized and thymectomy before or after 10 years of age, there was no significant difference in the rates of improved clinical status. Again, a larger group over longer follow-up may demonstrate differences in ultimate remission rates.

We exclusively utilize a left thoracoscopic approach, which we found provides good mediastinal and cervical exposure. Vigilant adherence to best practices of patient positioning and good thoracoscopic principles allows accurate dissection of peri-thymic tissue while minimizing risk to the patient. For example, the modified supine position and gentle insufflation allows lung isolation without selective intubation. Similarly, exclusive use of smart bipolar devices for hemostasis appears to reduce risk of thermal damage to the phrenic nerves. Meanwhile, a stereotyped dissection strategy minimizes anatomic confusion, reducing risk of damage to structures such as the innominate vein, and diminishing the probability of performing an incomplete thymectomy. Some advocate for a bilateral approach, but in the pediatric population, using an angled scope and optimizing retraction provides excellent visualization of the contralateral lobe (including any minor lobules) and the dissection comfortably carries to the contralateral pleura and to the superior horns, even if these extend into the neck.

Retrospective analysis of a heterogeneous population with a disease characterized by its variable fluctuating course is a challenging cohort from which to draw inferences about the study group as a whole. In particular, strong statements about the effectiveness of thymectomy in different subgroups cannot be made. Moreover, nothing can be said about optimal selection criteria—indications for surgery are not standardized, insufficient power exists among groups to discern probability of response, and too few patients have follow-up far enough to count most of those who will enter remission. There also remains an open question as to whether or to what degree thymectomy improves the probability of not progressing from ocular to more generalized symptoms.

These questions leave the larger question of surgical indication incompletely answered. Given the published literature, there is certainly no consensus regarding the role nor timing nor approach for thymectomy, and any patient with a diagnosis of JMG may be considered for operative management. The published evidence suggests that we can say with some confidence that thymectomy doubles or triples the probability of remission compared to medical treatment alone. This would be best validated with a multi-center randomized controlled trial in the pediatric JMG population. The experience of this larger group of patients with broad ranges of body size, age, and presentation lends much stronger support to the notion that a thoracoscopic approach can be done without leaving residual thymic tissue and with very low morbidity risk. Still, thoughtful consideration must be given to the potential for large blood loss and permanent nerve damage, the inherent learning curve for pediatric thoracoscopy, and the risk of persistent and worsening myasthenic symptoms even after surgery. Far greater numbers of patients are needed to detect rare risks and to identify those more likely to respond and achieve complete stable remission. To address this last concern, our intention is to develop a JMG registry, with this group forming the initial seed.

## Conclusion

Review of the largest series to date of thoracoscopic thymectomy for juvenile myasthenia gravis shows it is a safe and effective adjunct in the treatment armamentarium, even for ocular disease. Ongoing research will improve our understanding of rare risks, remission probability given different disease presentations, and overall outcomes over longer time scales. Development of a registry and continuing participation in multi-disciplinary efforts towards a pediatric randomized controlled trial are the next steps.
